# Molecular Aspects of Bone Resorption in β-Thalassemia Major

**DOI:** 10.22074/cellj.2016.3713

**Published:** 2015-07-11

**Authors:** Najmaldin Saki, Saeid Abroun, Fatemeh Salari, Fakher Rahim, Mohammad Shahjahani, Mohammadi-Asl Javad

**Affiliations:** 1. Health Research Institute, Research Center of Thalassemia and Hemoglobinopathy, Ahvaz Jundishapur University of Medical Sciences, Ahvaz, Iran; 2. Department of Hematology, Faculty of Medical Sciences, Tarbiat Modares University, Tehran, Iran; 3. Health Research Institute, Hearing Research Center, Ahvaz Jundishapur University of Medical Sciences, Ahvaz, Iran; 4. Department of Medical Genetics, School of Medicine, Ahvaz Jundishapur University of Medical Sciences, Ahvaz, Iran

**Keywords:** β-thalassemia, Bone Resorption, Bone Marrow, Osteoblasts, Osteoclasts

## Abstract

β-thalassemia is the most common single gene disorder worldwide, in which hemoglobin β-chain production is decreased. Today, the life expectancy of thalassemic patients is increased because of a variety of treatment methods; however treatment related complications have also increased. The most common side effect is osteoporosis, which usually occurs in early adulthood as a consequence of increased bone resorption. Increased bone resorption mainly results from factors such as delayed puberty, diabetes mellitus, hypothyroidism, ineffective hematopoiesis as well as hyperplasia of the bone marrow, parathyroid gland dysfunction, toxic effect of iron on osteoblasts, growth hormone (GH) and insulin-like growth factor-1 (IGF-1) deficiency. These factors disrupt the balance between osteoblasts and osteoclasts by interfering with various molecular mechanisms and result in decreased bone density.

Given the high prevalence of osteopenia and osteoporosis in thalassemic patients and complexity of their development process, the goal of this review is to evaluate the molecular aspects involved in osteopenia and osteoporosis in thalassemic patients, which may be useful for therapeutic purposes.

## Introduction

β-thalassemia is the most common single gene disorder worldwide, in which synthesis of the β-globin chain is decreased, leading to ineffective erythropoiesis (1, 2). Frequent blood transfusions increase the life expectancy of thalassemic patients. However osteopenia and osteoporosis are significant complications that contribute to morbidity of these patients. These two complications are observed in approximately 50% of β-thalassemia patients (3). Subsequent to this complication, bone fracture is noted in 36% of thalassemic patients (4). Osteopenia and osteoporosis are detected by the presence of reduced bone mineral density (BMD), reflecting decreased bone turnover (4).

In thalassemia, osteoporosis is a complicated process affected by several factors. The most important factors for osteoporosis in thalassemia included elayed puberty, diabetes mellitus, hypothyroidism, ineffective hematopoiesis with bone marrow hyperplasia, parathyroid gland dysfunction, toxic effect of iron on osteoblasts and deficiency of growth hormone/insulin-like growth factor-1 (GH/IGF-1). In general, decreased bone density and osteoporosis are the result of a disrupted balance between osteoblasts and osteoclasts (5, 6).

### Osteoblasts

Osteoblasts originate from mesenchymal stem cells (MSCs). Their production is increased by transforming growth factor-beta (TGF-β), basic fibroblast growth factor (bFGF) and bone morphogenetic protein (BMP) (7-9). These cells secrete macrophagecolony stimulating factor (M-CSF), granulocyte macrophage-CSF (GM-CSF), interleukin-1 (IL-1), IL-6 and TGF-β (10). These cytokines are also involved in bone formation because they release alkaline phosphatase (ALP), osteopontin, osteocalcin, collagen and fibronectin (4, 7, 11).

### Osteoclasts

These are multinucleate cells that originate from hematopoietic stem cells (HSC) under the effect of MCSF and the receptor activator of nuclear factor κB (NF-κB) ligand (RANKL), causing bone resorption by secretion of matrix metalloproteinase and cathepsin (4, 7, 12, 13).

**Fig.1:**
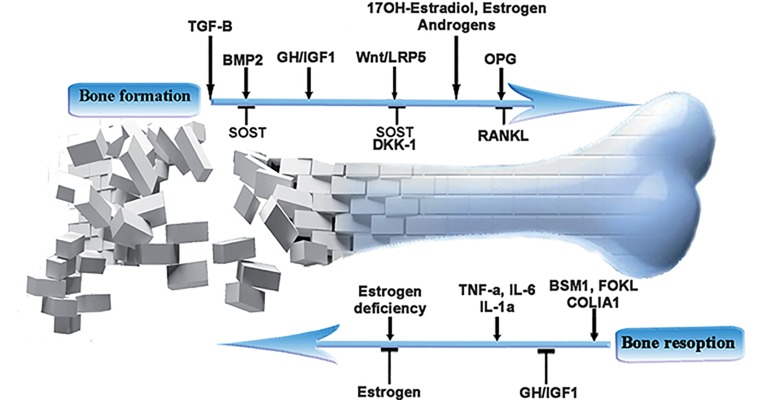
Important molecules involved in bone resorption in thalassemia. TGF-β; Transforming growth factor-beta, SOST; Sclerostin, DKK-1; Dickkopf, BMP2; Bone morphogenetic protein-2, GH; Growth hormone, IGF-1; Insulin-like growth factor-1, Wnt; Wingless related protein, LRP5; Low density lipoprotein (LDL)-related protein 5, OPG; Osteoprotegrin, RANKL; Activator of NF-κB receptor ligand, TNF-α; Tumor necrotic factor-alpha, IL-6; Interlukin-6, IL-1α; Interlukin-1 alpha and COLIA1; Collagen type I alpha 1 gene.

The RANK/RANKL cytokine system, parathyroid hormone (PTH), sex hormones (such as estrogen and testosterone), inflammatory cytokines, GH/ IGF-1, BMP2 protein as well as the wingless related protein/β-catenin (Wnt/β-catenin) signaling pathway and iron deposition in the bone marrow are the most common factors that affect the balance between these two cell types (14, 15) (Fig.1).

### Cytokine system

#### Osteoprotegrin/RANKL/RANK

This cytokine system is among the most effective mechanisms in bone reabsorption, which regulates bone density by several factors explained later (16) (Table 1). RANKL has three isoforms and its soluble form is secreted from osteoblasts. RANKL binding to its receptor on precursors and mature osteoclasts triggers the NF-κB pathway, resulting in differentiation, activation and survival of osteoclasts as well as bone turnover (17, 18). Osteoprotegrin (OPG), an antagonist of RANKL and member of the tumor necrosis factor (TNF) receptor superfamily, is secreted by osteoblasts and prevents the differentiation and activity of osteoclasts (14, 19-22).

In patients with thalassemia, toxicity of iron for osteoblasts along with endocrine effects increase RANKL and decrease OPG, thereby increasing the risk of osteoporosis (23, 24). In chronic diseases, disorders of iron deposition in body organs, endocrine disorders, malignant bone tumors and rheumatoid arthritis disrupt the OPG/RANKL balance which results in inhibition of bone reabsorption (25). Therefore, antibodies against OPG and RANKL (which have an inhibitory effect on osteoclasts) are used in the treatment of bone complications (4, 5).

**Table 1:** Overview of molecular mechanisms in bone resorption in thalassemia


Factors	Target (pathway/gene)	Role

PTH	PKA activation and down regulation ofOPG/RANKL ratio	Osteoclast activation
17OH-estradiol	Down regulation of JNK pathway onRANKL downstream	Reduction of osteoclast differentiation
Estrogen andtestosterone	Influence on OPG and RANKL mRNA	Up regulation of OPG/RANKL ratio
Estrogen	RUNX2 activation	Osteoblastic differentiation
	Fasl activation	Osteoclast apoptosis
IL-1α, IL-6, TNF-α	Initiation of the NF-κB pathway	Increased osteoclast differentiationand activation
TGF-β	Activation of Smad pathway andinduced RUNX2 production	Osteoblast differentiation
		IGF-1	Increased OPG, collagen type I, RUNX2,ALP production in HMSC	Osteoblastic differentiation
GH	Increased OPG	Inhibition of osteoclasto-genesis
	Increased BMP2	Induction of osteoblastic differentiation
BMP2	Elevated β-catenin level thatresults in CBF-α transcription	Osteoblasticdifferentiation
RUNX2	Wnt canonical pathway	Osteoblastic differentiation
Wnt/βcateninsignaling pathway	β-catenin stabilization	Osteoblastic differentiation
	Up regulation of OPG/RANKL ratio	Reduction of osteoclastic differentiation
DKK-1	Antagonizes canonical Wnt signaling byinhibiting LRP5/6 interaction with Wnt	Inhibition of osteoblastic differentiation
SOST	Inhibition of Wnt signaling	Inhibition of osteoblastic differentiation


PTH; Parathyroid hormone, PKA; Protein kinase A, TGF-β; Transforming growth factor-beta, SOST; Sclerostin, DKK-1; Dickkopf, BMP2; Bone morphogenetic protein 2, GH; Growth hormone, IGF-1; Insulin-like growth factor 1, Wnt; Wingless related protein, LRP5; Low density lipoprotein (LDL)-related protein 5, OPG; Osteoprotegrin, RANX2; Runt-related transcription factor 2, JNK; Janus kinase, ALP; alkaline phosphatase, HMSC; Human mesenchymal stem cells, NF-κB; Nuclear factor kappa B, RANKL; Activator of NF-κB receptor ligand, TNF-α; Tumor necrotic factor-alpha, IL-6; Interlukin-6 and IL-1α; Interlukin-1 alpha.

#### Inflammatory cytokines

Increased levels of inflammatory cytokines such as IL-1α, IL-6 and TNF-α (due to iron overload) (3, 10, 11) in the serum of thalassemic patients is inversely related to their bone density (26). The sepro-osteoclastogenic cytokines exert their effects predominantly via the OPG/RANKL system (3, 27).Thus, IL-6, IL-1α and TNF-α induce cyclooxygenase2 (COX2) and prostaglandin E2 (PGE2) which cause an increase in RANKL and a decrease in OPG, resulting in increased bone resorption (3, 19, 28). Moreover, these cytokines trigger the NF-κB and Janus kinase (JNK) pathways, ultimately increasing activation and differentiation of osteoclasts (10). IL-6 and TNF-α are also involved in pathogenesis of bone resorption in acute abdominal disease, rheumatoid arthritis and menopause-associated osteoporosis (14). Anti-TNF-α is used to improve bone metabolism in patients with rheumatoid arthritis (29).

#### Transforming growth factor-beta

Local reduction of TGF-β in the bone marrow is a likely risk factor of osteoporosis in patients with thalassemia (26). TGF-β is a type of receptor tyrosine kinase that phosphorylates Smad and induces the production of Runt-related transcription factor 2 (RUNX2) in mesenchymal precursors, resulting in osteoblastic differentiation *in vitro* (30, 31). TGF-β causes the death of osteoclasts by reducing the activity of C-JUN factorin the RANKL pathway (7, 32). Most cytokines have paracrine effects. The majority of studies have found no correlation between their circulating concentrations and bone resorption markers (33). Therefore, the best method is to analyze these cytokines in the tissues.

#### Bone morphogenetic protein 2

Expression of BMP2 is decreased in thalassemic patients with osteoporosis (34). BMP2 is acytokine from the TGF-β family involved in commitment of mesenchymal precursors to osteoblasts. Local production of BMP2 and TGF-β is associated with increased proliferation and differentiation of osteoblasts (20). BMP2 binding with serine/threonine kinase receptors on the cell surface phosphorylates Smad complex and activates transcription factors effective in osteoblastic differentiation such as RUNX2 and Ostrix (35-37). BMP2 also increases RUNX2 gene transcription by increasing the level of β-catenin, thereby differentiating mesenchymal cells to osteoblasts (38, 39). In addition, BMP2 plays a role in Ostrix activation and mesenchymal differentiation to osteoblasts by activating JNK and P38 factors and triggering the mitogenactivated protein kinase (MAPK) cascade (40-42). P53 inhibits BMP2 and exerts its inhibitory effect by suppressing Ostrix (43).

### Endocrine disorders

#### Growth hormone/insulin-like growth factor-1

Disruption of the GH/IGF-1 pathway is another mechanism in reducing bone density in thalassemic patients (5). GH stimulates the liver to secrete IGF- 1. Both hormones have an anabolic role in the bone marrow (16). IGF-1 is mainly released in the liver and GH in the anterior pituitary. In thalassemic patients, iron toxicity for the liver and anterior pituitary possibly reduce serum levels of IGF-1 and GH, respectively (16, 44). However, IGF-1 deficiency is prominently caused by hepatitis C virus (HCV) infection in these patients (45). IGF-1 increases the level of OPG, type I collagen, RUNX2 and ALP in human MSCs (hMSCs) (46) along with inducing the expression of Ostrix (via the MAPK pathway), which result in osteoblastic differentiation. Therefore, there is a positive relationship between the level of IGF-1 and BMD in thalassemic patients (47). According to research, reduction of IGF-1 plays a role in glucocorticoid-induced osteoporosis (48). GH stimulates the production of BMP and OPG, causing increased proliferation of osteoblasts and inhibition of osteoclast production, respectively (6, 8). GH deficiency has been reported in only 8% of β-thalassemic patients, and is mainly caused by iron overload. In contrast, IGF-1 production is impaired in 72% of patients (45). As a result, introduction of these hormones in thalassemic patients with hormone deficiency is recommended to prevent osteoporosis (3).

#### Parathyroid hormone

Long-term increase in PTH causes reduction of OPG/RANKL by activating osteoblastic protein kinase A (PKA), thereby increasing the activity of osteoclasts (49, 50).

#### Sex hormones

A reduced level of sex hormones in thalassemic men with hypogonadism and postmenopausal women can cause osteoporosis. In thalassemic patients, iron deposition in the anterior pituitary disrupts the release of sex hormones and delays puberty in 50% of patients (6, 15).

17OH-estradiol binds its alpha receptor on osteoclasts, decreasing the activity of JNK downstream of RANKL and inducing the production of OPG which results in inhibition of osteoclasts. Therefore, there is a strong correlation between 17OH-estradiol and serum concentrations of OPG and RANKL in thalassemic patients (6, 12, 18). Free estrogen and testosterone in thalassemic patients increase OPG mRNA and decrease RANKL (6, 8, 50). Estrogen also binds the alpha receptor on osteoblasts and osteoclasts, activating RUNX2 in osteoblasts and Fas ligand (Fasl) inosteoclasts, which results in increased osteoblastic differentiation and death of osteoclasts (21, 51-54). In addition, androgens and estrogens regulate resorption in bone by regulating cytokines secreted by osteoblasts and stromal cells such as IL1-α, IL-6, TGF-β and PGE2, which control the activity of osteoclasts through paracrine effects (33). Hormone therapy is an approach to prevent osteoporosis in thalassemic patients (3).

### Transcription factors

#### Runt-related transcription factor 2

RUNX2 is an early transcription factor in osteoblastic differentiation. This factor is decreased in thalassemic patients affected by iron deposition in the bone marrow (55). RUNX2 prevents differentiation of MSCs into adipocytes and chondrocytes through the wingless related protein canonical pathway (Wnt-1 pathway), and plays an important role in osteoblastic differentiation and bone formationby increasing BMP2 and induction of Ostrix expression (54, 56).

#### Ostrix

Reduction of Ostrix in thalassemia is associated with decreased BMD, and it is involved in the pathogenesis of osteoporosis in thalassemic patients (34). It is an essential transcription factor for differentiation of osteoblasts, which is activated by IGF-1, TGF-β and RUNX2, resulting in osteoblastic differentiation (40, 57).

#### Wnt/β-catenin signaling pathway proteins

Wnt proteins play an important role in regulating bone massby affecting osteoblastic maturation and activity. Wnt protein binding with Frizzled (Fz) receptor (a member of the G protein coupled receptors) and LDL related protein co-receptor (LRP) results in signal transduction, stability of β-catenin and its transfer to the nucleus, and eventual transcription of genes associated with osteoblastic differentiation (58-60). Wnt proteins increase OPG/RANKL through the β-catenin dependent canonical pathway (Wnt3a) in osteoblasts, causing an increase in osteoblastic differentiation and suppression of osteoclast production (17, 18, 51).

#### Dickkopf

DKK-1 is increased in serum of thalassemic patients who have osteoporosis (6), and is associated with reduced BMD in the lumbar vertebrae and end of the radius (59). Secreted molecular DKK-1 has a cysteine-rich domain at its carboxyl end, which binds LRP5/LRP6 co-receptors and inhibits Wnt binding with these cofactors, preventing osteoblastic differentiation as an antagonist of the canonical Wnt pathway (40, 61, 62). In addition, serum DKK-1 is increased in multiple myeloma (MM) patients with lytic bone lesions, menopause induced osteoporosis, Paget’s disease, glucocorticoid induced osteoporosis and estrogen deficiency (39, 63-65). Anti-DKK-1is used to treat bone loss in patients with MM (66).

#### Sclerostin

This factor is involved in the incidence of osteoporosis in thalassemic patients. Increased serum levels of this molecule are associated with reduced BMD in thalassemic patients (59). Sclerostin is a secretory molecule and product of the SOST gene which antagonizes LRP4, LRP5 and LRP6 co-receptors, resulting in inhibition of the Wnt canonical pathway and differentiation of osteoblasts (46, 62, 67) (Table 1). Sclerostin is also anantagonist of BMP2 (68), which is increased in MM (in which it is released by plasma cells) and in cancer-induced bone loss (69). Anti-sclerostin is used to treat menopause-related osteoporosis (59).

#### Genetic factors

Genetic factors play an important role in reduction of bone density and development of osteoporosis in thalassemia (Table 2). One factor is polymorphism in the SP1 region of the collagen type I alpha 1 gene (COLIA1), which has an incidence of 90% in thalassemic patients (14, 20). However, it plays no role in osteoporosis in sickle cell patients (70). Polymorphisms in the gene region of vitamin D such as BSM1 (in intron 8) and FOKL (in exon 2) are also associated with reduced bone density in patients with thalassemia and sickle cell anemia (71, 72).

**Table 2:** Genetic factors involved in osteoporosis in thalassemia


	COLIA-1 polymorphism	Down regulation in procollagen production

Genetic factors	BSM1 polymorphism	
	FOKL polymorphism	Down regulation in vitamin D absorption


COLIA-1; Collagen type 1 alpha 1.

## Discussion

Although increased osteoclastogenesis and in adequate osteoblastogenesis can cause an imbalance between bone formation and resorption with a possible decrease in BMD (7), osteoporosis in thalassemic patients is a complicated process influenced by multiple genetic and acquired factors. These factors not only affect the proliferation and activity of osteoblasts and osteoclasts, some such as gastrointestinal absorption disorder play an important role in providing the resources necessary for bone formation. In addition, the person’s age, nutritional and physiological conditions are effective in development of bone lesions. Therefore, the presence of a single factor cannot be considered a risk factor for osteoporosis in patients with thalassemia. Rather, a variety of factors must be examined together. In addition, as an osteoporotic skeleton has not been reported to be restored to healthy status in thalassemia, prevention of osteoporosis is of utmost importance in managing β-thalassemic patients. Considering the fact that bone resorption is caused by multiple factors, healthcare vigilance in these patients should be multifactorial. Prescription of calcium can provide adequate calcium levels during skeleton development and can increase bone mass (73-75). Moreover, prescription of vitamin D supplements plays an important role in this process (74, 76). Therefore, thalassemic patients should observe a proper diet as part of their preventive program. Early hormone replacement is the most effective strategy to prevent gonadal deficiency-induced bone loss (77, 78).

In clinical trials, novel therapeutic agents are very promising treatments for bone diseases. These agents include calcitonin and bisphosphonates. Calcitonin is a hormone secreted by the thyroid that inhibits osteoclastic activity. It causes bone pain relief and radiographic improvement after a one year administration in thalassemic patients (79). Also, bisphosphonates inhibit bone resorption and are as beneficial as estrogen, in preventing loss of bone mass (80). Anti-DKK-1 has a pivotal role in bone health for management of bone lesions in MM patients and is a novel therapeutic agent for these patients. However, the clinical trial role of anti-DKK-1 in thalassemic patients should be elucidated (81). Further studies are required to evaluate its effect as well as that of anti-sclerostin on β-thalassemic patients.

## Conclusion

This review has discussed a number of genetic and acquired factors that affect bone density in patients with thalassemia. Considering the above factors, hormone therapy, optimal transfusion (preventing precipitation of iron), calcium and vitamin D prescription can be effective in preventing bone lesions.

Given the high prevalence of musculoskeletal disorders in patients with thalassemia and considering the fact that osteopenia and osteoporosis are progressive disorders in these patients, early screening and preventive intervention are of utmost importance. In addition, annual bone density screenings in these patients is recommended. Although the factors mentioned in this article can be important to manage this process, further research in this field is needed.
